# The Frequency of Resurgery after Percutaneous Lumbar Surgery Using Dekompressor in a Ten-Year Period

**DOI:** 10.1155/2018/5286760

**Published:** 2018-10-09

**Authors:** Stephan Klessinger

**Affiliations:** ^1^Department of Neurosurgery, Nova Clinic Biberach, Eichendorffweg 5, 88400 Biberach, Germany; ^2^Department of Neurosurgery, University of Ulm, Albert-Einstein-Allee 23, 89081 Ulm, Germany

## Abstract

To prevent open surgical procedures, minimally invasive techniques, like Dekompressor (PLDD), have been developed. The absence of reherniation is an important factor correlating with clinical success after lumbar surgery. In this retrospective, observational study, the frequency of additional open surgery after PLDD in a long time retrospective was examined. The correlation between clinical symptoms and outcome was assessed, and the time between PLDD and open surgery was analyzed. Consecutive patients after PLDD between 2005 and 2007 were included. MacNab's outcome criteria were used to evaluate patient satisfaction. The need for additional open surgery of the lumbar spine, the period between Dekompressor and resurgery, and the treated levels were analyzed. In total, 73 patients were included in this study. The patients were seen one month after PLDD. The majority of patients (76.7%) had additional radicular pain. The most common level treated was L4-5 (58.9%). The follow-up time was longer than 5 years in 30.1% of the patients and longer than 10 years in 6.82%. The short-term success rate was 67.1%. Additional surgery was performed in 26.0% of patients, with 78.9% of the reoperations undertaken during the first year after PLDD. These patients had a statistically significant worse outcome (P = 0.025). Radicular pain was present in all patients with an early subsequent surgery, but only in 50% of patients with late surgery (P = 0.035). Significantly more patients with poor pain relief had radicular pain (P = 0.04). The short-term success rate was worsened by a resurgery rate of 26.0%. Subsequent surgery, a short time after PLDD, suggests that PLDD is not a replacement for open discectomy. Because patients with radicular pain had a worse outcome and more frequent resurgeries, whether radicular pain is an ideal indication for PLDD should be discussed.

## 1. Introduction

Lumbar radicular pain caused by disc herniation is often treated with open discectomy [1]. Its effectiveness has been demonstrated in controlled trials [2–4] and in long-term follow-up studies [5]. Minimally invasive techniques have been developed to prevent open surgery. The paucity of evidence supporting these minimally invasive techniques highlights the need for more data. Only limited evidence exists for Nucleoplasty and Dekompressor [6, 7].

Percutaneous lumbar disc decompression with Dekompressor (PLDD) uses the Archimedes' pump principle to mechanically remove a predetermined amount of disc material, reducing the pressure in the disc. The placement of the 1.5 mm cannula is similar to that used in a standard discography.

PLDD has been shown to be superior to conservative treatment [6, 8, 9] and has been associated with a low rate of complications [8]. However, limited outcome data are available. While preliminary studies revealed a favorable outcome [9–13], only two assessed clinical outcomes beyond one year [6, 10], and only one study measured the open surgery rate after PLDD [6]. The absence of reherniation is an important factor for patient satisfaction after discectomy [14]. The expected outcomes after revision surgery are less well defined than for primary discectomy [14]. Therefore, the number of subsequent surgeries after PLDD is of great importance.

It is important to find an ideal indication for PLDD. Unsuccessful conservative treatment is a prerequisite of any spine surgery. Patients with a clear indication for open discectomy are also not ideal candidates for a percutaneous technique. It seems that outcomes after microdiscectomy for contained herniations are worse than for sequestered herniations [15]. As such, Dekompressor has been devised for small contained disc herniations [1]. The idea is that the nucleus and annulus are in a closed system where the herniated part can move back towards the center after a decrease in volume. A contained disc is regarded as an important prerequisite to the success of PLDD [8].

Advantages of the Dekompressor system are the minimal damage to adjacent tissues [11]. Proponents of the system state that Dekompressor does not substitute disc degeneration [11]. Less pertinent scarring and less postoperative fibrosis may be expected [12].

The aim of this retrospective, observational study was to investigate the frequency of an additional open surgery after PLDD in a more than ten-year retrospective. The time between PLDD and open surgery was analyzed, and a correlation between the clinical symptoms and outcome was assessed.

## 2. Materials and Methods

In this retrospective observational study, the patient data were drawn from an electronic medical record system. PLDD was performed in a practice setting. Open disc surgery was performed in a general hospital.

Inclusion criteria were as follows: consecutive patients who underwent PLDD between January 2005 and December 2007. A history of pain for a minimum of 3 months was mandatory. Patients had either low back pain or radicular pain with or without a sensory loss. Patients with a lumbar spine surgery in their history were excluded.

**Figure 1:**
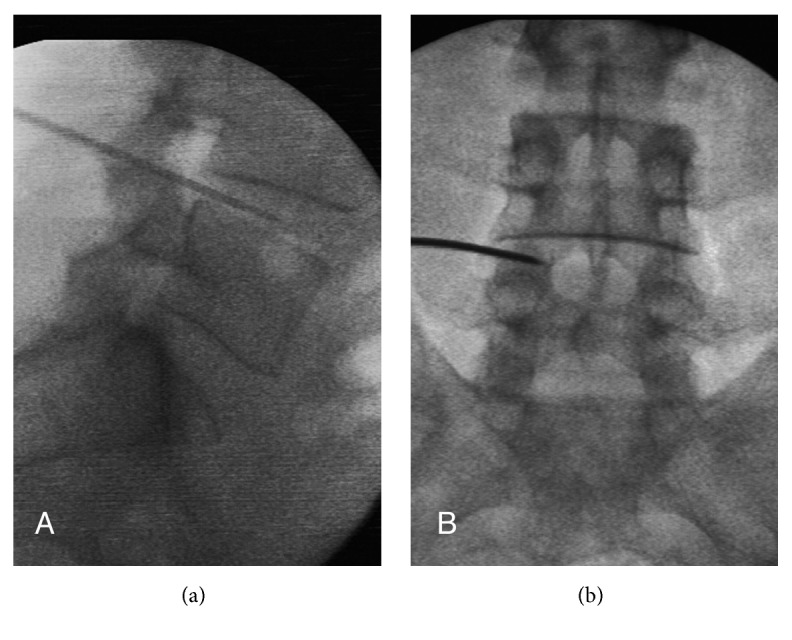
AP and lateral fluoroscopy image of the position of the PLDD wand at L4-5.

For PLDD, the 17-gauge Dekompressor probe (Stryker, Kalamazoo, Michigan) was used. Prophylactic antibiotics were administered prior to the procedure. A standard approach was used to place the introducer cannula with the stylet under fluoroscopic view intradiscally. The correct cannula placement was confirmed with anterior-posterior and lateral fluoroscopic images (Figure 1). The Dekompressor probe was advanced into the cannula and switched on. Disc material was harvested by moving the cannula along several passes intradiscally.

Every patient was seen in the practice personally one month after the operation for follow-up and later according to the complaints of the patient. A physician interview and a clinical examination were performed. A long-term follow-up of more than ten years was possible.

The age and gender of the patients, the treated levels, the follow-up time, and the pain characteristics (only lumbago or radicular pain with or without sensory loss) were evaluated. MacNab's outcome criteria [14] (1 = Excellent, no pain, no restriction of activity; 2 = Good, occasional pain; 3 = Fair, improved but handicapped by intermittent pain; 4 = Poor, no improvement) were used to measure the success after PLDD. The evaluation of the necessity of an additional open lumbar spine surgery was the focus of this study. The period of time between the PLDD and the resurgery and the treated levels and the symptoms of the patients (back pain or radicular complaints) were analyzed.

The Exact-Fisher-test was used to compare values of patients with substantial pain relief and poor pain relief. Welch's t-test was used to test the hypothesis that two populations had equal means. P < 0.05 was set as the threshold for interpreting the results as significant.

## 3. Results

Between January 2005 and December 2007, 86 patients were treated with PLDD. Because of spine surgery in their history, eleven patients were excluded. Two patients were lost to follow-up. Therefore, 73 patients were included in this study. The data of these patients are shown in Table 1. In total, 33 patients (45.2%) were women and 40 were men. The age of the patients was between 17 and 85 years, with the mean age being 48.9 years.

All patients had pain for more than three months (mean 6.6 months). Twenty-eight patients (38.4%) reported pain for more than one year before the treatment. Seventeen patients only had back pain. The majority of the patients (76.7%) had additional radicular pain. A sensory loss in the symptomatic leg was present in 43 patients (58.9%). No motor deficit was present.

The most common level treated was L4-5 (58.9%). Two levels (either L3-4-5 or L4-5-S1) were treated in 11 patients (15.1%). In 50.7% of patients the left side was symptomatic, while in 42.5 % of the patients, the right side was treated. Five patients (6.8%) were treated on both sides. No PLDD-related severe complications occurred.

The first follow-up examination one month after PLDD was mandatory for all patients. Further examinations were arranged according to the needs of the patients. This first follow-up was the only one in nine patients (12.3%). In 22 patients (30.1%), the follow-up was longer than 5 years, and in five patients (6.8%) it was longer than 10 years. The mean follow-up time was 35.6 months.

One month after the intervention, excellent results were achieved in 17 patients and good results, in 32 patients. Therefore, the short-term success rate was 67.1%. Subsequent surgery at the index level was necessary in 19 patients (26.0%). In these cases, the herniated disc fragment was removed, and a discectomy or a bony decompression of the spinal canal was performed.

Most reoperations (15 patients) had to be performed during the first year after PLDD (20.5% of all patients, 78.9% of all resurgeries). These patients (Table 2) had a statistically significant worse outcome (26.7% versus 75.0% satisfied patients, |t|=2.467, (*α*1 = 0.025)t(7)=2.365). Radicular pain was present in all patients with an early subsequent surgery, but only in 50% of patients with late surgery (P = 0.035). The mean time between PLDD and the additional surgery was at 10.8 ± 17.9 months (1–70 months).

Comparing the patients with excellent or good outcome (substantial pain relief) with the patients with poor pain relief (Table 3), significantly more patients with poor pain relief had radicular pain (91.7% versus 69.4 %, P = 0.04). As expected, the rate of resurgeries is higher if patients are not satisfied (50.0% versus 14.3%, P = 0.002).

## 4. Discussion

This retrospective observational study investigated the number of patients with a subsequent open surgery after PLDD. Patients with back pain only and patients with radicular pain were included. The short-term success rate was 67.1%; however, 26.0% of all patients had to undergo an additional surgery, most of them during the first year after PLDD. If resurgery was necessary, the primary outcome was worse compared to patients without surgery during follow-up. All patients with an early additional surgery had radicular pain. Patients with radicular pain had a worse outcome.

The short-term success rate is comparable with the few available studies from the literature. The recent study of McCormick et al. [6] found a 73% positive response after one year using a threshold of >50% improvement in NRS leg pain score and > 30% ODI improvement. This result builds on the other available studies with 6-to-24-month follow-up periods [9–13].

Also, the resurgery rate seems to be comparable with the only one study reporting these data. McCormick at al. [6] reported 36% additional surgery at the 8-year follow-up. All patients in his study had radicular pain. In the present study, the resurgery rate was 34% if only patients with radicular pain are taken into account. However, the patient selection in the McCormick study [6] and the present study is different. All patients in the McCormick study [6] were candidates for open discectomy. Therefore, it is concluded that PDLL had prevented spine surgery in 64% of cases. In the present study, no patient was a candidate for open surgery even though some of them had radicular pain or a sensory loss. However, no patient with a motor deficit or even bladder dysfunction was included. This means that the resurgery rate in the present study indicates additional surgery for a patient for whom conservative treatment was an alternative to PLDD. Recent studies found comparable resurgery rates for lumbar Nucleoplasty (18.7 %, [16]) and for cervical Nucleoplasty (19.5 % [17]).

Avoidance of surgery is an important goal in reducing morbidity and mortality [6]. From the data of this study it remains unclear whether PLDD can achieve this objective. It is also worth considering whether radicular pain is a good indication for PLDD. Generally, patient selection appears to be extremely important in the efficacy of PDD [8, 18]. The best results may be obtained when the disc herniation is contained [8, 18] and is limited to a single level [18]. For Ong et al. [8], the exact role of PLDD in the treatment of radicular pain is still up for debate, but PLDD should not be abandoned. Lee [19] concludes that, in spite of the lack of the evidence, the Dekompressor may be worth trying in patients with leg pain and contained disc herniations prior to open discectomy because the Dekompressor is easy to apply, is relatively safe, and causes less injurious to the disc. In contrast to the studies of Ong et al. [8] and Lee [19], the present study shows that radicular pain is an inferior indication compared to low back pain.

With an early resurgery, it was suspected that the indication for PLDD was too generous. Another explanation for early resurgery was the risk of acute herniation after the puncture of the disc with the 17-gauge needle. The risk of acute herniation is dependent on the needle diameter. The most vulnerable site is the inner annulus [20]. All patients with an early additional surgery had radicular pain. Patients with radicular pain had worse outcomes compared to patients with back pain only. As a consequence, a good indication might be a patient with low back pain without radicular symptoms with a contained disc. Trying Dekompressor in the first instance risks an additional surgery with lower success rates [1, 21]. This study suggests that Dekompressor is unable to replace open surgery.

There are limitations to this study. This audit is retrospective and observational and therefore does not represent a high level of evidence. However, the resurgery rate is an important factor for the outcome.

## 5. Conclusions

At first sight, a satisfied patient level of 67% seems to be a good result. However, this short-term result is significantly worsened due to a resurgery rate of 26.0%. Subsequent surgery a short time after PLDD suggests that PLDD is not a replacement for open discectomy. A contained disc herniation causing low back pain without radicular pain appears to be a good indication for Dekompressor. Because patients with radicular pain had a worse outcome and more frequent resurgeries, whether radicular pain is an ideal indication for PDLL should be discussed. Further studies are needed to compare the outcome and rate of subsequent surgery in patient populations with and without radicular symptoms to find the ideal indications for PLDD.

## Figures and Tables

**Table 1:** Patient characteristics.

All Patients	
n	73

Age (years)	
mean	48.9 ± 13.4
min-max	17–85
Female	33 (45.2%)
Level	
L2-3	2 (2.7%)
L3-4	4 (5.5%)
L3-4-5	3 (4.1%)
L4-5	43 (58.9%)
L4-5-S1	8 (11.0%)
L5-S1	13 (17.8%)
Side	
left	37 (50.7%)
right	31 (42.5%)
both	5 (6.8%)
Radicular Pain	56 (76.7%)
Sensory Loss	43 (58.9%)
Follow-up (months)	
mean	35.6 ± 40.2
min-max	1–132
Macnab's outcome criteria	
mean	2.2 ± 1.0
substantial pain relief (1+2)	49 (67.1%)
Additional surgery at index level	19 (26.0%)
Period until surgery (months)	
mean	10.1 ± 17.1
min-max	1–70

**Table 2:** Patient characteristics dependent on the time of resurgery and significant differences between these two groups.

	All Patients with Resurgery	Resurgery		Significance
		during first year	later	
n	19	15 (78.9%)	4 (21.1%)	

Radicular pain	17 (89.5%)	15 (100.0%)	2 (50.0%)	**P = 0.035**

Macnab's outcome criteria				
mean	2.9 ± 0.8	3.1 ± 0.8	2.3 ± 0.5	**|t|=2.467, (** **α** **1 = 0.025)t(7)=2.365**
substantial pain relief (1+2)	7 (36.8%)	4 (26,7%)	3 (75.0%)	
Period until surgery (months)				
mean	10.8 ± 17.9	3.0 ± 2.3	40.3 ± 20.8	**|t|=3.588, (** **α** **1 = 0.025)t(3)=3.182**

**Table 3:** Patient characteristics dependent on the outcome and significant differences between these two groups.

	All Patients	Result		Significance
		substantial pain relief	poor pain relief	
n	73	49 (67.1%)	24 (32.9%)	

Radicular pain	56 (76.7%)	34 (69.4%)	22 (91.7%)	**P = 0.04**

Additional surgery	19 (26.0%)	7 (14.3%)	12 (50.0%)	**P = 0.002**

## Data Availability

The data used to support the findings of this study are available from the corresponding author upon request.
